# Genome-wide association identifies key loci controlling blackberry postharvest quality

**DOI:** 10.3389/fpls.2023.1182790

**Published:** 2023-06-07

**Authors:** T. Mason Chizk, John R. Clark, Carmen Johns, Lacy Nelson, Hamid Ashrafi, Rishi Aryal, Margaret L. Worthington

**Affiliations:** ^1^ Department of Horticulture, University of Arkansas, Fayetteville, AR, United States; ^2^ Department of Horticultural Science, North Carolina State University, Raleigh, NC, United States

**Keywords:** firmness, red drupelet reversion, GWAS, polygalacaturonase, pectin methylesterase (PME), *Rubus*, polyploidy

## Abstract

**Introduction:**

Blackberry (*Rubus* subgenus *Rubus*) is a soft-fruited specialty crop that often suffers economic losses due to degradation in the shipping process. During transportation, fresh-market blackberries commonly leak, decay, deform, or become discolored through a disorder known as red drupelet reversion (RDR). Over the past 50 years, breeding programs have achieved better fruit firmness and postharvest quality through traditional selection methods, but the underlying genetic variation is poorly understood.

**Methods:**

We conducted a genome-wide association of fruit firmness and RDR measured in 300 tetraploid fresh-market blackberry genotypes from 2019-2021 with 65,995 SNPs concentrated in genic regions of the *R. argutus* reference genome.

**Results:**

Fruit firmness and RDR had entry-mean broad sense heritabilities of 68% and 34%, respectively. Three variants on homologs of polygalacturonase (PG), pectin methylesterase (PME), and glucan endo-1,3-β-glucosidase explained 27% of variance in fruit firmness and were located on chromosomes Ra06, Ra01, and Ra02, respectively. Another PG homolog variant on chromosome Ra02 explained 8% of variance in RDR, but it was in strong linkage disequilibrium with 212 other RDR-associated SNPs across a 23 Mb region. A large cluster of six PME and PME inhibitor homologs was located near the fruit firmness quantitative trait locus (QTL) identified on Ra01. RDR and fruit firmness shared a significant negative correlation (*r* = -0.28) and overlapping QTL regions on Ra02 in this study.

**Discussion:**

Our work demonstrates the complex nature of postharvest quality traits in blackberry, which are likely controlled by many small-effect QTLs. This study is the first large-scale effort to map the genetic control of quantitative traits in blackberry and provides a strong framework for future GWAS. Phenotypic and genotypic datasets may be used to train genomic selection models that target the improvement of postharvest quality.

## Introduction

Fresh-market blackberries are growing in popularity in the United States, and year-round availability is heavily dependent on imports during the winter months. From 2019 to 2022, U.S. fresh-market blackberry imports have sharply risen by about 52% (United States Department of Agriculture, Economic Research Service, 2023) to meet growing demand from consumers. Soft-fruited blackberries that are shipped over long distances suffer from numerous postharvest issues, including leakage, mold, berry softening, deformation, weight loss, and discoloration through red drupelet reversion (RDR). RDR is a postharvest disorder that causes individual drupelets on fully ripe blackberries to ‘revert’ from shiny black to a red, underripe appearance. This disorder presents a significant risk to producers because the United States Department of Agriculture Agricultural Marketing Service (USDA-AMS) has established a grading standard that recommends the rejection of blackberry lots if 10% of the berries are affected by RDR or 5% are categorized as severe (United States Department of Agriculture, Agricultural Marketing Service, 2018). These standards align with the opinions of consumers, who strongly prefer blackberries with minimal RDR (Threlfall et al., 2020).

Many factors impact the incidence and severity of RDR, including mechanical damage during harvest and shipping, climate conditions, time of harvest, and excessive nitrogen fertilization (Pérez-Pérez et al., 2018; Edgley et al., 2019b; Edgley et al., 2019c). At the chemical level, RDR is characterized by the delocalization of anthocyanins from vacuoles, leading to degradation and color change (Edgley et al., 2019d; Kim et al., 2019). RDR has been disproportionately associated with soft-fruited cultivars (Salgado and Clark, 2016; Armour et al., 2021), suggesting that RDR may be a visual indicator of poor fruit texture observable under certain postharvest conditions. Thus, it may also be possible to identify QTL that are pleiotropic for both traits. Several studies have documented the broad range of phenotypic diversity for fruit texture and RDR present across the University of Arkansas (UA) System Division of Agriculture Fruit Breeding Program blackberry germplasm (Salgado and Clark, 2016; Threlfall et al., 2016; Segantini et al., 2017; Armour et al., 2021). Fruit texture was once considered an intractable trait, but cultivars released in recent decades have improved shipping potential. Landmark cultivars like ‘Navaho’ and ‘Chester Thornless’ (Clark, 2005) were some of the first to have true shipping potential, thus expanding the potential for a global blackberry industry. Major improvements in texture quality have coincided with growth in blackberry popularity and an increasing emphasis on improving fruit texture for fresh-market breeding programs (Clark and Finn, 2011; Finn and Clark, 2011). Several UA blackberry breeding selections possess a distinctly firm-textured phenotype described as ‘crispy’. These cultivars have less RDR compared to non-crispy genotypes (Salgado and Clark, 2016). Great strides in improving blackberry shipping potential over decades of breeding selection demonstrate the heritability of firm texture and RDR resistance among the UA System population. Still, the genetic control of these traits and their association remains unexplored.

Advances in computational biology, high throughput sequencing, and the increasing availability of powerful statistical packages for polyploid plants have enabled genome-wide association studies (GWAS) to investigate marker-phenotype associations in autopolyploid plant species (Bourke et al., 2018). The initial objective of such studies was to identify the physical genomic positions of large-effect quantitative trait loci (QTL) influencing traits of interest in breeding populations. A natural downstream objective of these studies was the identification of causal variants or loci which are in a state of high linkage disequilibrium (LD) with causal variants. Such variants may then be implemented in marker-assisted selection (MAS) strategies to cull undesirable seedlings and select optimal parental combinations. In the context of perennial crops, like blackberry, where the fruit is often not observed until the second year of growth, MAS may greatly improve the rate of genetic gain in breeding programs (Foster et al., 2019). For example, identification of loci influencing fruit quality in blackberry may provide breeders with a path to select genotypes of superior texture in less than half the time of traditional phenotyping methods.

The blackberry germplasm in the UA System Fruit Breeding Program consists predominately of autotetraploid materials. Thus, it is important to consider an association model that accounts for polyploid allele dosage. Driven partly by declining costs and technological advances, a growing community of polyploid researchers have produced open-source tools that will enable more robust GWAS pipelines in recent years. The R package, updog, provides researchers with the ability to flexibly genotype polyploids using next-generation sequencing (NGS) datasets that suffer from variable read depth, sequencing error, allele bias, and overdispersion (Gerard et al., 2018). The GWASpoly package has made association mapping of autopolyploids more accessible by providing functions capable of testing multiple hypothetical dominance models (Rosyara et al., 2016). GWASpoly also allows users to build models that consider both random marker effects and fixed population structure (Q + K model). In tetraploid blueberry, polyploid GWAS models detected greater numbers of significant marker-trait associations than diploid models (Ferrão et al., 2018). Lastly, the ldsep package contains utilities for pairwise estimation of linkage disequilibrium (LD) in polyploids (Gerard, 2021) which may provide an important contextual understanding of newly identified QTLs and their potential associations with nearby genes.

Functional understanding of the blackberry genome is in its infancy, but the recent assembly and annotation of a diploid blackberry reference genome (Brůna et al., 2022) has established a strong foundation for association mapping. The assembled genome, *Rubus argutus* cv. ‘Hillquist’ (298 MB), is an important donor of the primocane-fruiting trait to the UA System germplasm. A novel algorithm integrating genomic, transcriptomic, and cross-species protein evidence was used to predict a total of 38,503 protein-coding genes, of which 72% were functionally annotated. With available NGS strategies, previously described software packages, and an annotated reference genome, conditions are ideal for performing a GWAS that investigates complex quantitative traits like fruit texture and postharvest quality in blackberries.

## Materials and methods

### Plant materials and harvest

The GWAS panel consisted of 300 UA System blackberry breeding selections and publicly available fresh-market blackberry cultivars. These cultivars and selections were maintained in 6-m plots at the UA System Fruit Research Station (FRS) in Clarksville, AR. The FRS site is located at 35°31’5”N and long. 93°24’12”W, in USDA hardiness zone 7b, on Linker fine sandy loam. All plots were maintained with varying degrees of standard cultural inputs such as training/tipping primocanes to a hedgerow training system, annual dormant pruning, irrigation, chemical weed, disease, and pest control. The diversity panel was evaluated in 2019, 2020, and 2021 for firmness and red drupelet reversion. For each genotype, blackberries were harvested at the shiny-black stage into 500-mL clamshells on two harvest dates per year, with at least one week between harvest dates. All fruit was harvested in June and July between 10:00 a.m. and 5:00 p.m. to encourage RDR occurrence as reported by Edgley et al. (2019a) and Armour et al. (2021). After rain events, a minimum period of 24 hours was allowed to pass before harvest resumed. Fruit containing defects, such as ruptured or discolored drupelets, were discarded before placing in storage. Clamshells were filled just below the lid and placed directly into a portable cooler chilled by ice packs until they could be transported. In 2020 and 2021, clamshells of harvested fruit were placed on a custom-built steel table for thirty minutes, with a vibrating surface that produced 2 mm of displacement and a frequency of 10 Hz. This treatment was intended to simulate shipping conditions that lead to RDR by replicating the findings of Pérez-Pérez et al. (2018). Samples were stored for seven days at 5°C and 90% relative humidity. Prior to obtaining images, clamshells were removed from refrigeration and allowed to reach room temperature.

### Red drupelet reversion and image analysis

After seven days of cold storage, the fruit was removed from cold storage and returned to room temperature. The fruit was arranged in a single layer, with spacing between fruit, on a green plastic cutting board and photographed in a photo box (LimoStudio 16” x 16” Table Top Photo Photography Studio Lighting Light Tent Kit in a Box, AGG349; Las Vegas, NV) using a Canon EOS Rebel T3 (Tokyo, Japan) mounted directly above the staging board. In 2019, a standard US quarter dollar was included in each image as a size reference. In 2020 and 2021, an X-Rite ColorChecker Classic Mini (Grand Rapids, MI) was included in each image and used as a size reference. Digital images were analyzed using the ShinyFruit app, which is maintained on GitHub (https://github.com/mchizk1/ShinyFruit).

### Fruit firmness

Following image analysis, ten randomly selected berries per clamshell were assessed for firmness using a Stable Micro Systems TA.XT.Plus Texture Analyzer (Texture Technologies Corporation, Hamilton, MA). A fruit compression test was performed by placing individual berries horizontally on a flat surface using a cylindrical plane probe of 7.6 cm diameter at a rate of 2 mm.s^-1^ with a trigger force of 0.02 N. The probe traveled 5 mm after first contact, and the peak force (N) was recorded as berry firmness.

### BLUP and heritability analysis

Best linear unbiased predictions (BLUPs) of fruit firmness and RDR were calculated for each genotype across years and harvest replicates with the help of the lme4 (Bates et al., 2015) in (R Core Team, 2022). Because of imbalanced replication inherent to the large phenotypic dataset, harmonic means for year (*y*) and replicate (*r*) were used in entry-mean heritability analyses. Genotypic variance (σ^2^_g_), genotype by year variance (σ^2^_gy_), and residual variance (σ^2^) components were estimated with the lme4 model and used to calculate broad sense heritability as follows:


$$H_{per-plot}=\;\frac{\sigma_{g}^{2}}{\sigma_{g}^{2}+\;\sigma_{gy}^{2}\;+\;\sigma^{2}}$$

Hentry−mean= σg2σg2+ σgy2y + σ2yr

Broad sense heritability per-plot indicates the level of heritability expected when making selections in the absence of replication. When heritability is calculated on an entry mean basis, the researcher assumes that heritability is considered in the context of replication. This often increases heritability estimates due to reduced the effects of environmental variance components. All custom R scripts used to produce BLUPs, harmonic means, variance estimates, and heritabilities are available through a GitHub repository for repeatability (https://github.com/mchizk1/UA_Fruit_Breeding).

### Genotyping with capture-seq

Young leaf tissue was collected from each of the 300 genotypes in the GWAS panel, and DNA was extracted using a modified cetyltrimethylammonium bromide (CTAB) extraction protocol following Porebski et al. (1997). Quantification of DNA was performed using the Qubit dsDNA assay kit (Invitrogen, Carlsbad, CA) and samples were standardized to 40 ng/µl. Capture-Seq genotyping was performed at RAPiD Genomics (Gainesville, FL) with 35,054 custom biotinylated 120-mer probes distributed across the *R. argutus* genome. The majority of the probes were designed to target genic regions, including a number of genes implicated in cell wall metabolism in other fruits such as polygalacturonase (PG), pectinmethylesterase (PME), pectin lyase, and expansin. DNA libraries were sequenced with Illumina HiSeq to achieve an average of 5.14 million 150 bp paired-end reads per sample (**Supplementary Table 1**).

### SNP calling and quality filtering

Raw sequencing data was cleaned, trimmed, and aligned to the *R. argutus* genome (Brůna et al., 2022) using MOSAIK (Lee et al., 2014). Initial diploid variant calling was performed using Freebayes (Garrison and Marth, 2012). The VCF file was filtered using VCFtools (Danecek et al., 2011) to produce a file with biallelic markers with minor allele frequency ≥ 0.01 and read depths ranging between three and 750 per sample. The filtered diploid VCF file was recalled estimating tetraploid allele dosage using the multidog function in updog (Gerard et al., 2018) and SNPs with greater than 5% estimated error rate were discarded.

### Population structure

A Q matrix was generated in STRUCTURE (Pritchard et al., 2000) using the population admixture model and proposed K values ranging from two to eight with a burnin period of 10,000 and 20,000 Markov chain Monte Carlo replications. An appropriate K value was selected based on the ΔK statistic reported by Evanno et al. (2005).

### Genome-wide association

Association analyses were conducted using the GWASpoly package (Rosyara et al., 2016) in R. Both fruit firmness and RDR BLUPs were associated with the filtered set of tetraploid SNPs under the ‘Q+K’ model. The K matrix was constructed internally using the leave-one-chromosome-out (LOCO) method and the STRUCTURE-generated Q matrix was included to account for fixed admixed population clusters across individuals. Additive, simplex dominance (1-dom), and general models of gene action were considered. Minor allele frequency and maximum genotypic thresholds were set to 0.05 and 0.95, respectively. Marker scores were tested internally using an α=0.05 significance threshold and the ‘M.eff’ method. Using this method, *LOD* significance thresholds of 5.45, 5.45, 4.38, and 5.38 were used for additive, general, simplex alternative allele dominance, and simplex reference allele dominance models, respectively. The general model employed by GWASpoly places no restraints on the effects of dosage levels tested. QQ-plots were constructed from association results to compare observed marker scores against nominal *P*-values.

### Linkage disequilibrium

The average decay of *r^2^* between SNPs was estimated internally in GWASpoly using a maximum number of 10,000 SNP pairs and eight degrees of freedom for spline plotting. Using the ldsep R package (Gerard, 2021), Lewontin’s *D’* was estimated between a subsample of SNPs in each chromosome, with a minimum of 100 kb spacing between sampled SNPs. Heatmaps of chromosomal linkage disequilibrium (LD) matrices were constructed using the LDheatmap R package (Shin et al., 2006).

### Candidate gene mining

For significant SNPs that did not reside on an annotated texture gene, genomic flanking regions of 1 Mb were investigated for the nearest biologically likely candidate gene. For markers that were in high LD with one another, candidate genes were collectively considered in the flanking regions of each marker. Seventy-two percent of the 38,503 predicted protein-coding genes in *R. argutus* were functionally annotated as described by Brůna et al. (2022). Genes with functions related to cell wall disassembly in other plant species were considered promising candidates.

## Results

### Phenotypic data

**Figure 1:**
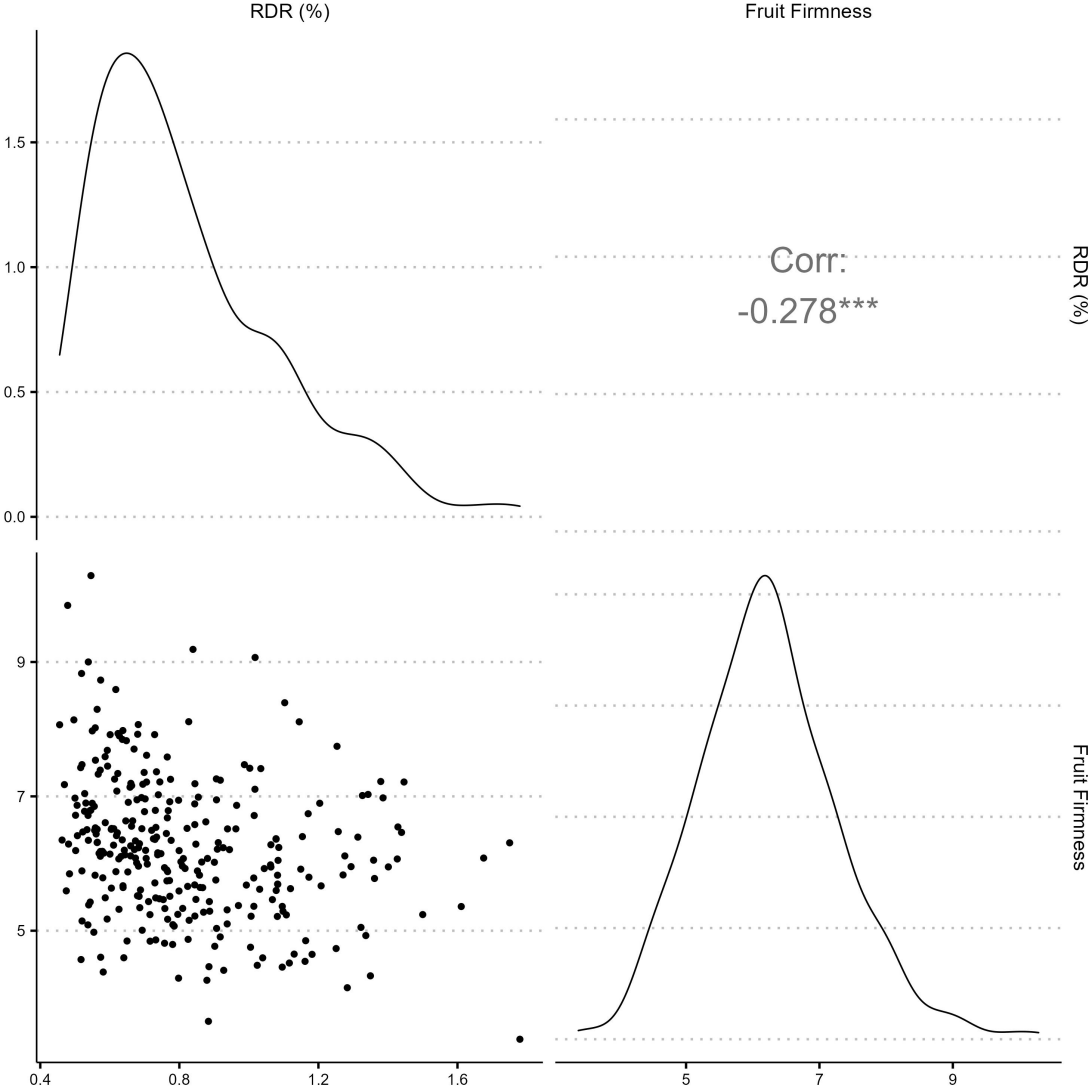
Phenotypic distributions and correlation of red drupelet reversion (RDR) measured by ShinyFruit and fruit firmness (N) best linear unbiased predictions across 300 tetraploid blackberry genotypes observed in 2019, 2020, and 2021.

Texture analysis was performed on 1,374 samples from 300 blackberry genotypes (**Supplementary Table 1**). Fruit firmness BLUPs were normally distributed (**Figure 1**) around a mean of 6.21 N, with values ranging from 3.38 - 10.29 N (**Supplementary Table 1**). The broad-sense entry-mean heritability fruit firmness was 0.68 (**Table 1**). Image analysis for RDR was performed on 1,370 samples from 299 blackberry genotypes. RDR values were right-skewed (**Figure 1**), with values ranging from 0.46% to 2.2% reverted pixels (**Supplementary Table 1**). The broad-sense entry-mean heritability of RDR was 0.31 (**Table 1**), less than half as heritable as fruit firmness. BLUPs for fruit firmness and RDR were weakly but significantly correlated (*r* = - 0.28).

**Table 1:** Variance components and broad-sense heritability of blackberry firmness and red drupelet reversion (RDR) across 2019-2021.

		Fruit firmness^vi^	RDR^vii^
Variance components	σ^2^_g_ ^i^	1.524	1.371
	σ^2^_gy_^ii^	0.323	0.405
	σ^2iii^	1.699	7.781
Heritability (%)	H_per-plot_^iv^	0.430	0.143
	H_entry-mean_ ^v^	0.677	0.335

i genotypic variance.

^ii^genotype by year variance.

^iii^residual error variance.

^iv^Heritability estimated in the context of single plot observations.

v Heritability estimated in the context of replication.

^vi^Peak force measured by 10 mm compression (N).

^vii^Percent RDR measured by ShinyFruit R package.

### Genotypic data

**Figure 2:**
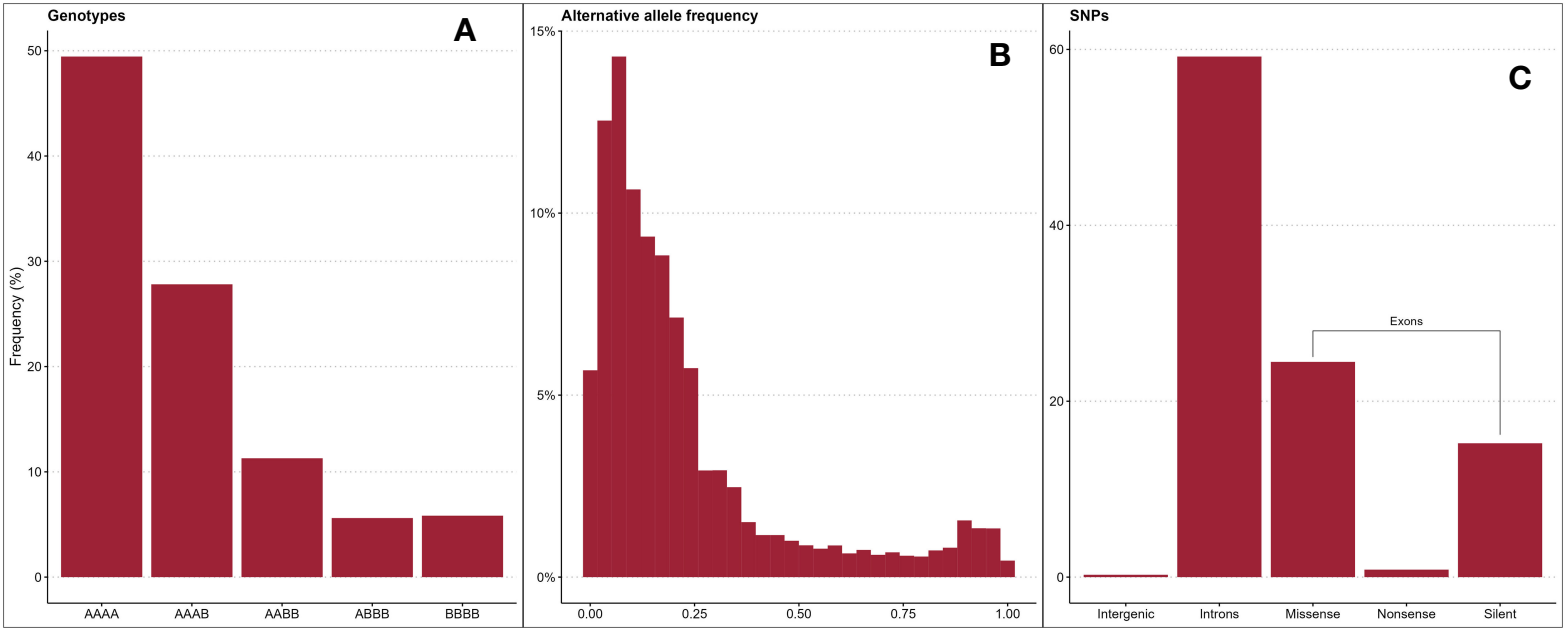
Distributions of blackberry genotypic frequencies across all loci in all individuals, where “A” represents the reference allele and “B” represents the alternative allele **(A)**, alternative allele frequencies **(B)**, and variant class frequencies **(C)** of 65,995 SNPs used in association mapping.

**Figure 3:**
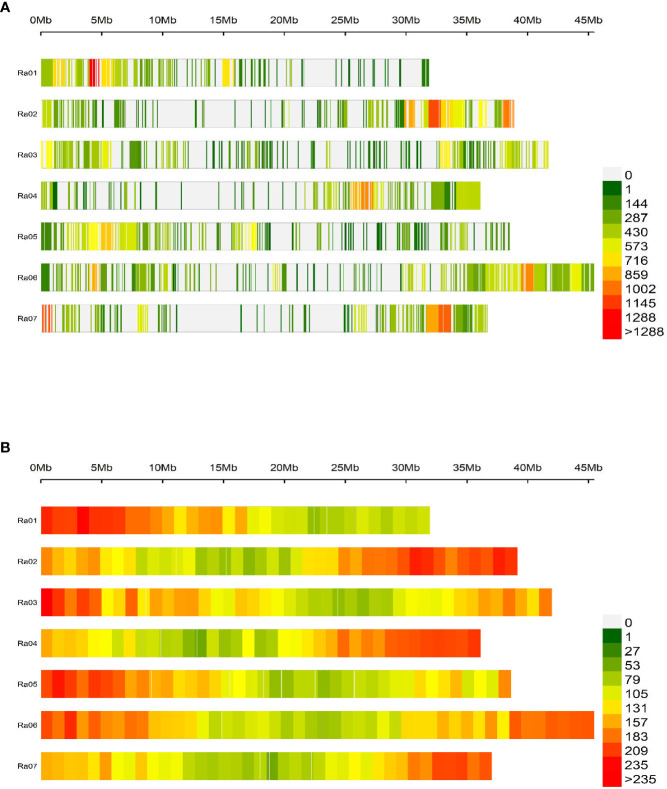
Distribution of **(A)** 65,995 SNPs and **(B)** 37,377 predicted genes across the seven chromosomes contained in the *R. argutus* reference genome. Color bins are assigned based on the number of SNPs or genes contained in a 1 Mb region.

In total, 124,564 biallelic SNPs were discovered in initial variant calling across a larger panel of 502 genotypes. After removing genotypes not evaluated in this study and SNPs of unacceptable quality or frequency, 65,995 SNPs were used in association analyses. The average read depth for these 65,995 SNPs across all genotypes was 216 (**Supplementary Figure 1**; **Supplementary Tables 1, 2**). The filtered SNP set had an average alternative allele frequency of 0.25 (**Figure 2A**, **Figure 2B**). Intergenic regions were avoided in the probe design stage, so over 99% of quality-passing SNPs were in annotated genic regions (**Figure 2C**), and 41% were located on coding sequences (CDS). About 60% of CDS polymorphisms were predicted to be missense mutations, and 2% were predicted to be nonsense mutations. The filtered SNP set was somewhat unevenly distributed across the R. argutus genome (**Figure 3A**), but closely mirrored gene density (**Figure 3B**).

### Population structure and LD

**Figure 4:**
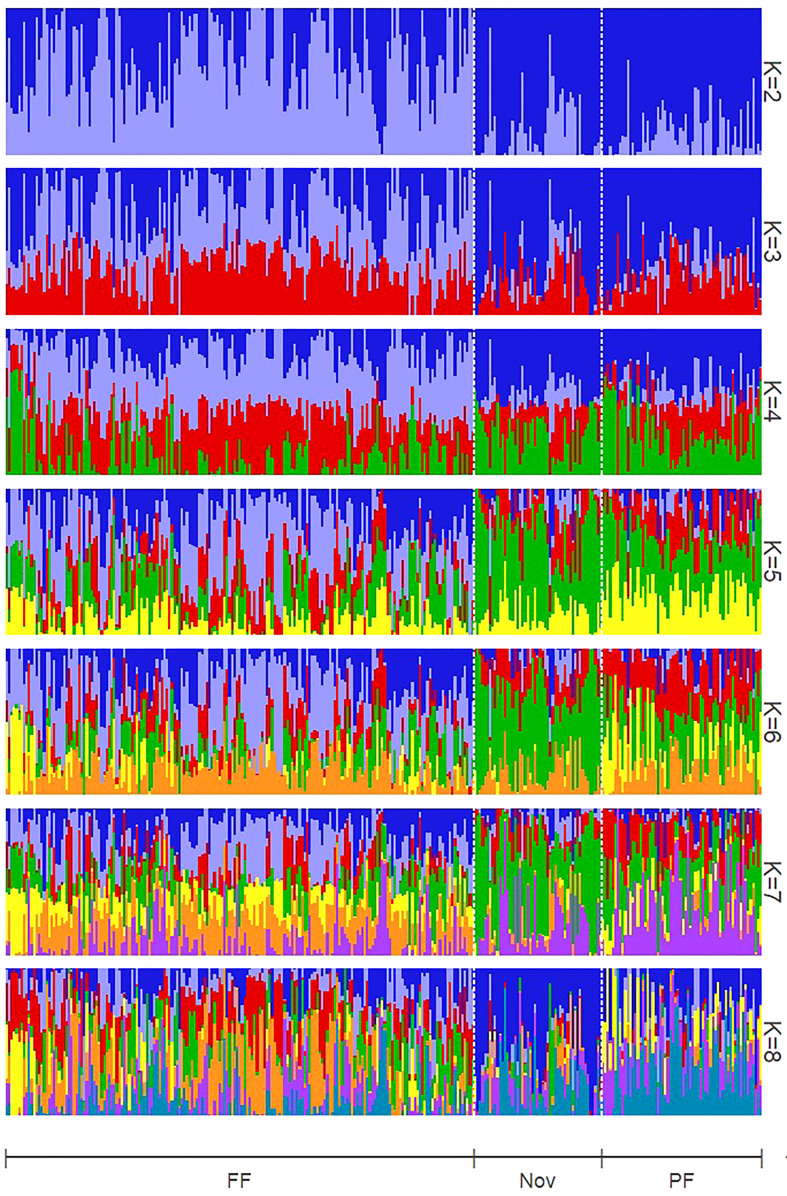
Structure estimation of admixed subpopulation proportions assuming numbers of subpopulations (K) ranging from two to eight. FF, floricane fruiting (non-primocane fruiting); Nov, Novels (brachytic dwarfing habit); PF, Primocane fruiting. K = 6 was optimal following Evanno et al. (2005).

**Figure 5:**
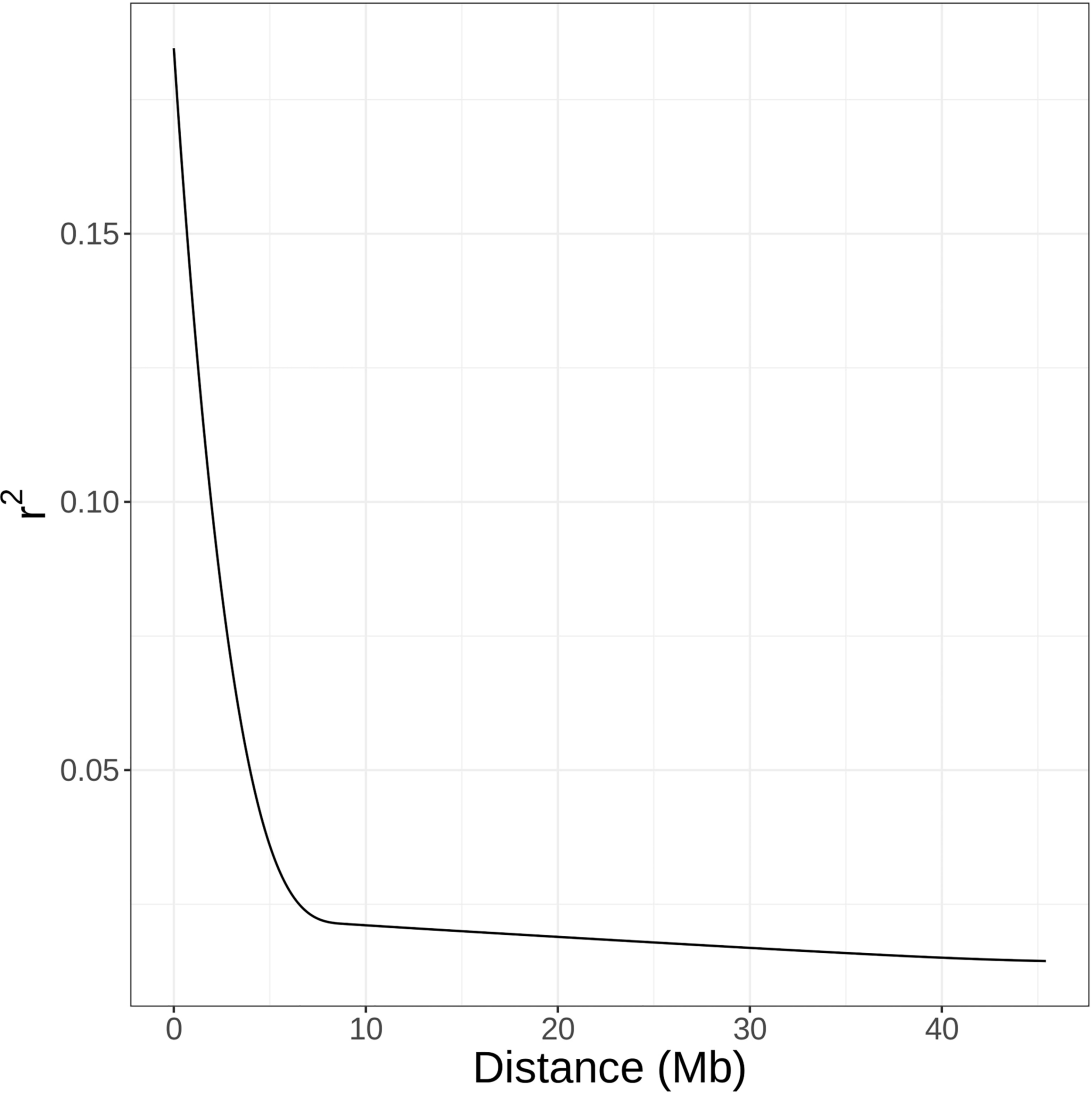
Average decay of linkage disequilibrium as estimated by squared correlation coefficients (r^2^) between SNPs across the whole blackberry genome.

**Figure 6:**
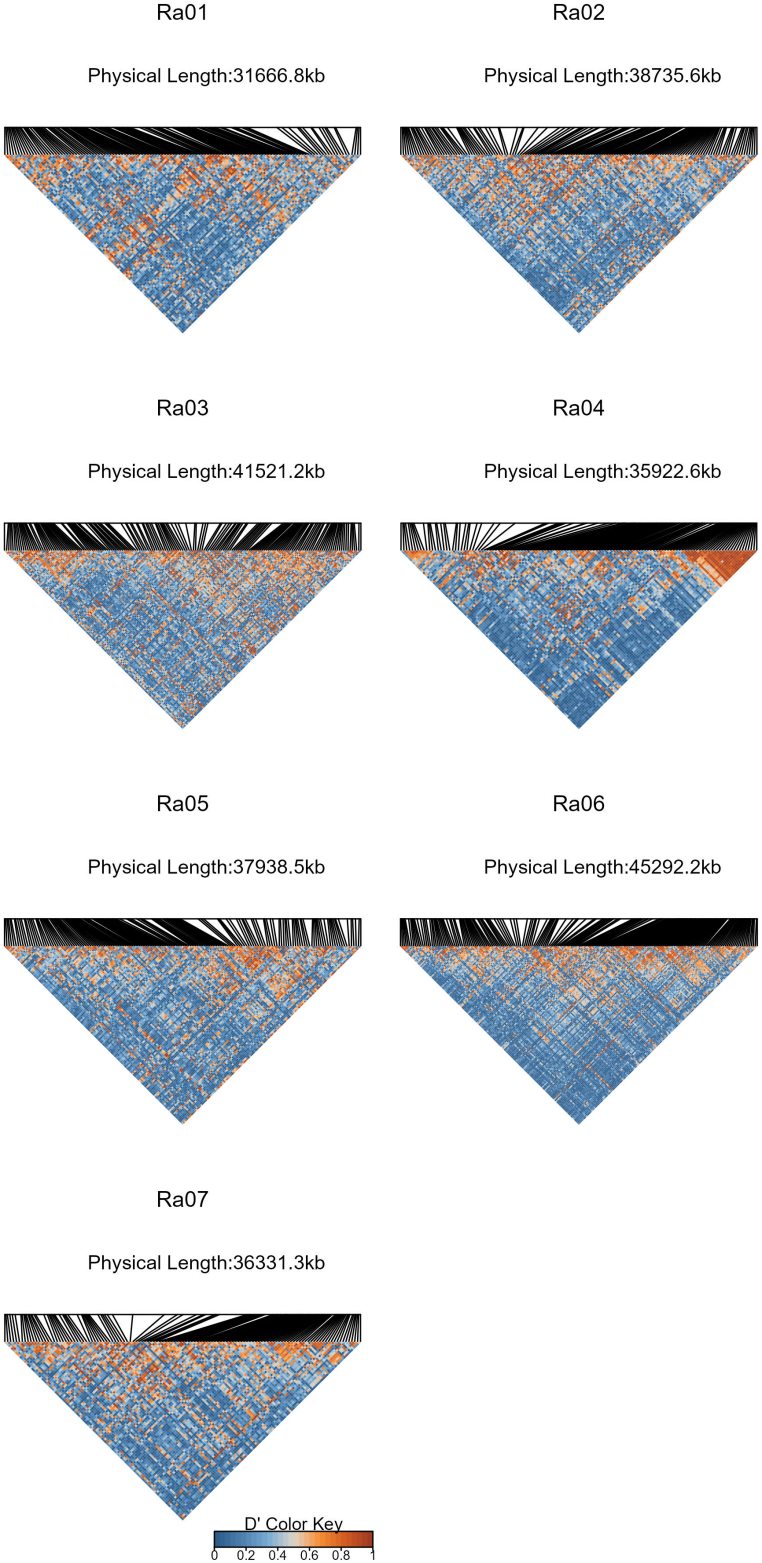
Heat maps of absolute values of Lewontin’s D’ (Lewontin, 1963) estimated between SNP subsets in each chromosome of the *R. argutus* blackberry reference genome. SNPs were subsampled to have a minimum spacing of 100 kb.

By comparing successive STRUCTURE simulations, *K* = 6 had the largest *ΔK* value of 93.4 (**Supplementary Figure 2**), and apparent differences were visible between the known subpopulations in the UA germplasm (**Figure 4**). These three subpopulations are primocane fruiting, floricane fruiting, and ‘novel’ or brachytic dwarf genotypes. Although crosses occur between these populations, they each align with specific market niches. Thus, a Q matrix assuming six admixed subpopulations was generated and used to reduce type I errors due to population structure in the GWAS model. LD associated with the physical linkage between SNPs largely decayed within 5 Mb (**Figure 5**), but LD decay varied across the genome, with the most apparent high-LD block located at the distal end of chromosome Ra04 (**Figure 6**).

### Genome-wide associations

**Figure 7:**
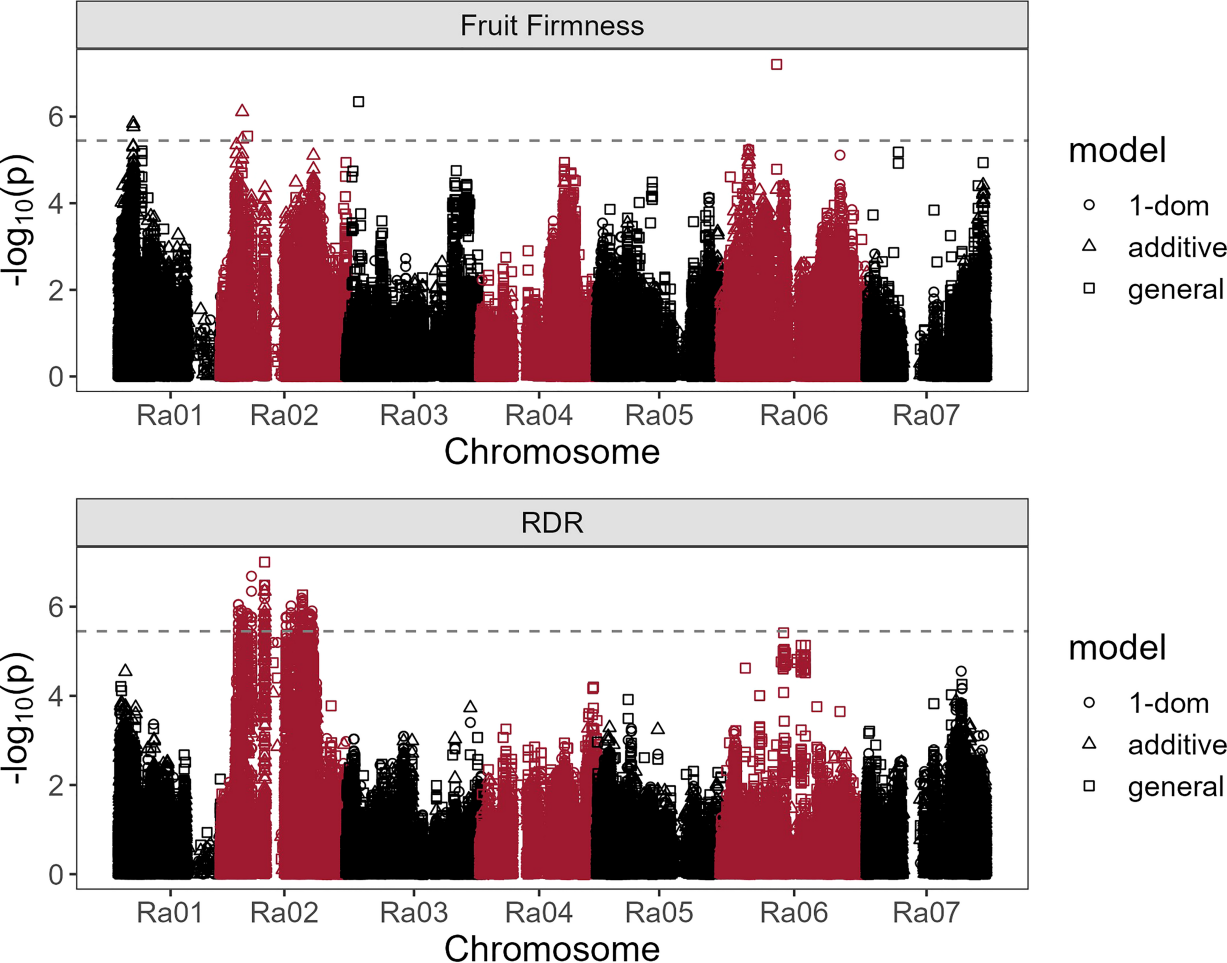
Manhattan plots of blackberry firmness and red drupelet reversion (RDR) associations with 65,995 SNPs under additive, general, and simplex-dominance (1-dom) models. The dashed line indicates the lowest significance threshold used between tested models at α = 0.05 using the GWASpoly M.eff method.

Fruit firmness was significantly associated with seven SNPs across chromosomes Ra01, Ra02, Ra03, and Ra06 (**Table 2**; **Figure 7**). All significant associations were identified under either additive or general dominance models, with general markers having the highest *LOD* scores. In a multi-SNP fruit firmness prediction model, Ra01:4352940_T/G, Ra02:6321387_T/C, and Ra06:17273160_A/G explained about 27% of all observed phenotypic variance in fruit firmness. The most significant association (*LOD* = 7.21) was observed under the general model at 17,273,160 bp on chromosome Ra06. Chromosomal QQ-plots of fruit firmness marker scores generally showed a gradual inflation in observed *LOD* scores compared to nominal values on chromosomes Ra01, Ra02, and Ra03 (**Supplementary Figure 3**).

**Table 2:** SNP-trait associations and candidate genes for blackberry firmness and RDR detected across 2019-2021.

Trait	Chromosome	Model	Position	Ref	Alt	*LOD*	*r^2^* (%)^i^	Variant type	Gene	Annotation and *Arabidopsis* homolog^ii^
Fruit firmness	Ra01	additive	4352940	T	G	5.84	5.27	Intron variant	Ra_g890	Pectinesterase/pectinesterase inhibitor PPE8B; At3g43270
		additive	4422023	T	G	5.77	5.36	67,850 bp ds^iii^	Ra_g890	Pectinesterase/pectinesterase inhibitor PPE8B; At3g43270
	Ra02	additive	6321370	C	T	6.11	11.40	Silent	Ra_g5252	Glucan endo-1,3-beta-glucosidase 7; At4g34480
		additive	6321387	T	C	5.48	10.48	Missense	Ra_g5252	Glucan endo-1,3-beta-glucosidase 7; At4g34480
		general	8025922	G	T	5.55	12.70	NA		
	Ra03	general	3633404	G	A	6.34	8.76	833,124 bp us^iv^	Ra_g10675	Glucan endo-1,3-beta-D-glucosidase; At2g43670
	Ra06	general	17273160	A	G	7.21	11.47	Silent	Ra_g27483	Probable polygalacturonase; At3g15720
RDR	Ra02	1-dom-ref	5150596	G	A	6.05	8.26	Missense	Ra_g5108	Probable polygalacturonase; At3g15720
		1-dom-ref	5150747	G	T	5.63	7.66	Missense	Ra_g5108	Probable polygalacturonase; At3g15720
		1-dom-ref	6320015	C	A	5.52	7.44	Missense	Ra_g5252	Glucan endo-1,3-beta-glucosidase 7; At4g34480
		general	13296902^v^	C	A	7.00	11.13	Intron variant	Ra_g5949	2-oxoglutarate-dependent dioxygenase; AT2G36690.2
		1-dom-ref	21494838	C	T	5.55	7.64	Missense	Ra_g6621	Endo-1,3;1,4-beta-D-glucanase; At3g23600
		1-dom-ref	21495058	A	T	6.02	8.33	Missense	Ra_g6621	Endo-1,3;1,4-beta-D-glucanase; At3g23600
		1-dom-ref	24484457	T	C	5.76	7.97	Missense	Ra_g7012	Expansin-like A1; At3g45970
		1-dom-ref	24485338	T	C	5.38	7.41	Missense	Ra_g7012	Expansin-like A1; At3g45970
		1-dom-ref	24485380	C	A	5.38	7.41	Missense	Ra_g7012	Expansin-like A1; At3g45970
		1-dom-ref	25139841	T	C	5.46	7.47	Missense	Ra_g7125	Expansin-A20; At4g38210
		1-dom-ref	25140011	G	A	5.76	7.97	Nonsense	Ra_g7125	Expansin-A20; At4g38210
		1-dom-ref	25140131	T	A	5.76	7.97	Missense	Ra_g7125	Expansin-A20; At4g38210
		1-dom-ref	25140272	A	C	5.81	7.96	Missense	Ra_g7125	Expansin-A20; At4g38210
		1-dom-ref	26764458	T	C	5.38	7.51	Missense	Ra_g7381	Glucan endo-1,3-beta-glucosidase A6; At3g07320
		1-dom-ref	26765115	G	C	5.76	7.97	Missense	Ra_g7381	Glucan endo-1,3-beta-glucosidase A6; At3g07320
		1-dom-ref	26765139	A	G	5.38	7.51	Missense	Ra_g7381	Glucan endo-1,3-beta-glucosidase A6; At3g07320
		1-dom-ref	26765333	A	G	5.38	7.51	Missense	Ra_g7381	Glucan endo-1,3-beta-glucosidase A6; At3g07320
		1-dom-ref	26765357	G	A	5.76	7.97	Missense	Ra_g7381	Glucan endo-1,3-beta-glucosidase A6; At3g07320
		1-dom-ref	26765634	T	G	5.76	7.97	Missense	Ra_g7381	Glucan endo-1,3-beta-glucosidase A6; At3g07320
		1-dom-ref	27713275	A	G	5.38	7.41	Missense	Ra_g7552	Probable pectate lyase 8; At3g07010
		1-dom-ref	27715029	C	T	5.38	7.51	Missense	Ra_g7552	Probable pectate lyase 8; At3g07010
		1-dom-ref	27715116	A	G	5.76	7.97	Missense	Ra_g7552	Probable pectate lyase 8; At3g07010

i Phenotypic r^2^ estimates were calculated based on single marker prediction models.

^ii^The SwissProt and Araport11 databases were interrogated for descriptions of gene homologs and the *Arabidopsis* homolog identifier following Brůna et al., 2022.

^iii^Downstream from candidate end position.

^iv^Upstream from candidate start position.

v Not on or near any annotated gene with known role in cell wall disassembly. All significant SNPs for fruit firmness are listed, but only 22 of 212 significant RDR SNPs were listed. All but one of these 22 SNPs with significant RDR associations were nonsynonymous mutations on genes with previously documented roles in cell wall modification/degradation. The full list of significant genes associated with RDR is available in **Supplementary Table 2**.

Red drupelet reversion was significantly associated with 220 SNPs (**Supplementary Table 2**), all of which were scattered across a 23.3 Mb region on chromosome Ra02. Notably, the RDR association analysis did not identify any of the same SNPs associated with fruit firmness, but 68 of the significant RDR markers were located on homologs of genes commonly associated with fruit texture (**Table 2**). Two hundred and eighteen RDR markers were discovered using the 1-dom ref model. However, the general model identified Ra02:13296902_C/A as the strongest marker for predicting RDR (*LOD* = 7.00; partial *r^2 = ^* 0.11) and was located on an intron region of Ra_g5949. As observed in fruit texture analysis, QQ-plots show gradual inflation of marker scores compared to nominal values on chromosome Ra02 (**Supplementary Figure 4**).

### Potential candidate genes

Four of the seven SNPs associated with fruit firmness were positioned directly on gene homologs associated with fruit texture, including PG, PME, and glucan endo-1,3-β-glucosidase (**Table 2**). On chromosome Ra06, a single silent PG variant on 17,273,160 bp (Ra_g27483) had the highest *LOD* score, an *r^2^* of 11%, and was located less than 200 kb away from two additional PG homologs. The highest scoring marker on Ra01 was on an intron region of the PME homolog, Ra_g890. The 1 Mb region flanking this variant contained a large cluster of six other PME and PME inhibitor homologs, one PG, and eight other texture-related homologs. Two significant SNPs were found on the same β-glucosidase homolog on chromosome Ra02, with the variant on 6,321,387 bp functioning as a missense mutation. The SNP located at 3,633,404 bp on Ra03 was about 833 kb away from two glucan endo-1,3-β-glucosidase homologs. No additional candidates for fruit texture were identified in regions immediately surrounding the SNP located on 8,025,922 bp on Ra02.

Most of the 220 significant SNPs associated with RDR on Ra02 were in a state of very high LD with one another (**Supplementary Figure 5**), even though not all of them were physically linked. Such widespread LD, combined with the large number of significant SNP associations, expanded the candidate gene search window to a region that covered much of chromosome Ra02. Nonsynonymous RDR SNPs located on texture homologs were reported in **Table 2**, and all other candidates neighboring the 23 Mb, high-LD region were compiled in **Supplemental Table 2**. Sixty-eight RDR-associated markers were located on just seven texture-related homologs including one PG, one pectin lyase, two glucan endo-1,3-β-glucosidases, two expansins, and one β-glucanase. All seven putative texture homologs contained at least one nonsynonymous mutation. Notably, all nonsynonymous mutations on texture homologs were detected under the 1-dom tetraploid dominance model. Among the seven polymorphic texture homologs, the expansin-like Ra_g7125, was the only putative texture gene to contain a nonsense mutation. The PG homolog Ra_g5108 contained a nonsynonymous mutation with the highest *LOD* value (6.05) for RDR. The strongest SNP for predicting RDR (*LOD* = 7.00; partial *r^2 ^= *0.11) was located on an intron region of Ra_g5949, which is a homolog of 2-oxoglutarate-dependent dioxygenase 19 in rice. This SNP is not located within 1 Mb of any homologs with documented roles in cell wall disassembly.

## Discussion

### Insights into genotypic datasets

GWAS has been used to discover marker-trait associations in many fruit crops, but to date, there have been no studies on the genetic control of quantitative traits of economic importance in blackberry. In this study, we used Capture-Seq genotyping and GWASpoly (Rosyara et al., 2016) to identify genetic regions associated with fruit firmness and RDR in tetraploid, fresh-market blackberries. Similar approaches have already been implemented to successfully map traits related to productivity and fruit quality in tetraploid blueberries (Ferrão et al., 2018; Ferrão et al., 2020). In the present study, we attempted to improve mapping resolution by analyzing a set of 65,995 SNPs highly concentrated in genic regions (**Figure 2C**). Nearly half of these SNPs were in coding exon regions, increasing the likelihood of observing SNP associations on or near causal variants. Average read depths exceeded 150x and produced high-quality SNP dosage calls using the updog R package (Gerard et al., 2018). LD among this population decayed over relatively large distances (**Figure 5**), which is likely the result of breeding activity and relatedness among individuals compared to more diverse populations or landraces (Rahimi et al., 2019). The large LD block located at the end of Ra04 may also provide evidence of an important domestication gene and a corresponding selective sweep in this region (Kim and Stephan, 2002).

### Insights into phenotypic datasets and heritability

In general, blackberry fruit firmness and RDR appear to be highly polygenic traits associated with numerous small-effect QTL. However, several important underlying trends were observed that must be considered in the interpretation of marker-trait association. The moderate heritability (*H* = 68%) of fruit firmness in our population (**Table 1**) suggests that environmental factors play an important role in determining firmness after storage; however, these estimates are not very different from those reported in strawberry (Shaw et al., 1987) or blueberry (Cellon et al., 2018) breeding populations. Environmental factors contributing to variation in blackberry fruit firmness include the addition of water, N, or Ca, pressure from fungal pathogens, respiration rates, and pests such as spotted wing drosophila (Prange and DeEll, 1997; Lee et al., 2011).

With a broad-sense heritability of 0.32 across three years of data collection, RDR appears to be particularly subject to environmental influence, supporting the work of Edgley et al. (2019c); Edgley et al. (2019b); Edgley et al. (2019a). In addition, the right-skewed nature of the RDR phenotypes observed is likely to mask many true differences in postharvest quality towards the lower end of the distribution. In other words, genotypes with very low susceptibility to RDR probably performed no differently than those mildly prone to reversion due to the nature of the distribution. Mechanical damage during harvest and shipping increases the incidence and severity of red drupelet reversion (Edgley et al., 2019a). However, berries in this study were harvested with much more care than is typical in commercial production. The vibration treatment implemented in 2020 and 2021 following Pérez-Pérez et al. (2018) did not increase RDR relative to 2019. Future studies should focus on treatments to increase RDR in to levels more comparable with commercial conditions. Observed RDR was weakly explained by variation in fruit firmness (*r* = -0.28), which follows previously reported relationships between these traits (Salgado and Clark, 2016; Armour et al., 2021). The weakness of this correlation could indicate the presence of other factors contributing to RDR severity, such as anthocyanin content or composition. Armour et al. (2021) identified differences in cyanadin-3-rutinoside content among blackberry genotypes, also noting that ‘Osage’ had higher cyanadin-3-rutinoside content and low RDR. Other authors have provided strong evidence that the color changes brought on by RDR result from anthocyanin degradation (Edgley et al., 2019d; Kim et al., 2019). Following the methods outlined in this study, future GWAS should explore anthocyanin content to search for more heritable sources of resistance to RDR.

### Fruit firmness and its genetic associations

Based on a moderate broad sense heritability estimate (*H* = 0.68) and the presence of multiple significant marker-trait associations on four out of seven chromosomes (Ra01, Ra02, Ra03, and Ra06), the genetic control of fruit firmness appears to be highly polygenic. Major pathways for fruit softening in other species are well-characterized in the literature. The two genes most frequently implicated in fruit softening are PG (Crookes and Grierson, 1983; Peace et al., 2005; Toivonen and Brummell, 2008) and PME (Marangoni et al., 1995; Wen et al., 2013; Xue et al., 2020), which act in unison to degrade pectin in the middle lamella. PME catalyzes the hydrolytic de-esterification of homogalacturonan (HG) regions of pectin molecules, while PG preferentially hydrolyzes ɑ-1,4-D galacturonan linkages in HG regions of pectin molecules, leading to softening through a loss of cell adhesion. Hence, both enzymes are necessary for the biologically programmed process of softening to occur. Significant marker-trait associations for fruit firmness were detected on homologs of both PG (Ra06) and PME (Ra01), although both variants were synonymous mutations and are, therefore, not expected to impact protein function. These significant associations on chromosomes Ra01 and Ra06 reside near larger surrounding clusters of linked PG and PME genes in flanking regions. Six PME, one PG, and eight additional homologs with documented roles in cell wall disassembly were located within 1 Mb of the significant SNP on Ra01. Two additional PG homologs were less than 200 kb away from the significant SNP on Ra06. The causal variants on these chromosomes are likely associated with at least one of these nearby homologs. In peach, a cluster of PG genes is responsible for the inheritance of melting flesh in peach (Callahan et al., 2004), and variable gene copy number is an important source of texture diversity (Gu et al., 2016). Slightly elevated LD levels surrounding the Ra01 locus (**Figure 6**) seems to indicate this gene cluster may have experienced high selection pressure through breeding activities. Fruit firmness in blackberry could be governed by gene clusters similar to the one reported in peach, and the effects of gene copy number have not yet been explored.

Three significant fruit firmness SNPs were located on Ra02. The most important of these (*LOD* = 6.11) was a silent mutation on the glucan endo-1,3-β-glucosidase homolog Ra_g5252. Like PG, β-glucosidase degrades fruit texture by cleaving pectin molecules. Instead of hydrolyzing HG regions, β-glucosidase degrades the ‘hairy’ arabinogalactan side chains of the pectin macromolecule (Devries et al., 1983), which are not targeted by PG. Therefore, β-glucosidases are thought to promote a more complete degradation of middle lamellar pectin molecules. A single glucan endo-1,3-β-glucosidase homolog (Ra_g5252) on Ra02 contained two SNPs significantly associated with fruit texture. This gene is the only candidate shared between fruit firmness and RDR association analyses, and it contains the only nonsynonymous mutation associated with fruit firmness discovered in this study (**Table 2**). The significant RDR-associated SNP on Ra_g5252 was positioned only 1,355 bp from the closest fruit firmness SNP. Prupe.8G098000, a homolog Ra_g5252 found in peach, is a β-glucosidase that is upregulated by treatment with 1-MCP (Qian et al., 2022), and the authors have speculated that it may be important in strengthening cell walls. Although the role of β-glucosidases in fruit texture has been well documented in several species (Gerardi et al., 2001; Konozy et al., 2012; Ortiz Araque et al., 2019), few studies have demonstrated genetic variation β-glucosidase and its potential connection to phenotypic variation for fruit texture. In sweet cherry, four β-glucosidase homologs were located on a fruit firmness QTL in chromosome four (Cai et al., 2019). In addition to their ability to modify pectin, glucan endo-1,3-β-glucosidases have a documented role in disease suppression (Kebede and Kebede, 2021), which may have the knock-on effect of preserving texture quality. It was more challenging to identify likely candidate genes for significant SNPs located at 8,025,922 bp on Ra02 and 3,633,404 bp on Ra03 were, as no known texture homologs appeared anywhere in the 1 Mb flanking regions. Interestingly, the SNP on 8,025,922 bp of Ra02 explained the most variation in fruit texture (12%) under a single marker prediction model, even though it was not located near any known texture homologs. This association could be the result of widespread LD between this SNP and the large, scattered group of texture genes associated with RDR (**Supplemental Figure 5**).

### RDR associations and the effects of LD

An inspection of QQ-plots identified a gradual inflation of *LOD* scores on some chromosomes compared to expected nominal values (**Supplemental Figure 4**). This trend is especially apparent on chromosome Ra02 for both RDR and fruit firmness association analyses. In SNP sets with large numbers of intergenic variants, these higher-than-expected *LOD* scores can be an indication of false discovery resulting from confounding factors that were not modeled, such as population structure or other covariates. In contrast, our models accounted for relatedness and covariance by including Q and K matrices, and 99% of our SNPs were positioned on intron and exon regions. Schork et al. (2013), who referred to this phenomenon as ‘enrichment’, reported how overrepresentation of SNPs in genic regions, such as introns and exons may result in higher numbers of significant *P*-values consistent with true polygenic effects. If this is indeed true in our own association analyses, the high number of postharvest QTL observed in this study would be consistent with findings in strawberries (Cockerton et al., 2021), which 24 QTL for fruit firmness widely distributed across the genome. In the case of blackberry, RDR may be a highly quantitative trait controlled by numerous low-effect genes on chromosome 2. Thus, future molecular breeding strategies for improving postharvest quality in blackberry should prioritize genomic prediction models over MAS approaches.

Despite the heavily right-skewed distribution of the RDR dataset, the genetic associations with this trait uncovered an interesting phenomenon on chromosome Ra02. Many widely distributed SNPs were significantly associated with RDR, and nearly all of them were in high LD (**Supplementary Figure 4**). Each of these SNPs explained nearly equivalent levels of phenotypic variance, with *r^2^* values ranging from 7.41% to 8.33% (**Table 2**). It is worth noting that these high LD SNPs were as far apart as 22 Mb, meaning this trend is not occurring due to physical linkage, but may instead be the result of heavy selection pressures. The relative importance of specific candidate genes may be obscured by the fact all seven RDR-associated genes containing nonsynonymous SNPs were in LD. Therefore, it could be true that only either one, a few, or indeed all RDR-associated genes may be necessary in their contributions to RDR resistance and postharvest quality.

Several gene candidates appear to be especially noteworthy. The SNP with the highest *LOD* score for RDR was a missense mutation on Ra_g5108, a polygalacturonase homolog (**Table 2**). The expansin-like gene, Ra_g7125, contained the only significantly associated nonsense mutation, suggesting probable loss of function. Powell et al. (2003) reported that simultaneous transgenic suppression of PG and expansin genes in tomatoes synergistically prevented fruit softening. The high LD between nonsynonymous mutations present on Ra_g5108 and Ra_g7125 suggests that a similar multigenic model of control for RDR could be possible. The missense mutation at 6,320,015 bp of Ra02 was on Ra_g5252, the same β-glucosidase homolog identified in the fruit firmness association, providing a potential link between fruit firmness and RDR. In addition to PG, expansin, and β-glucosidase, significant associations with RDR were found on a pectate lyase (PL) homolog, Ra_g7552. In strawberry, transgenic antisense inhibition of PL has reduced fruit softening during ripening (Jiménez-Bermúdez et al., 2002).

### Genetic architecture of postharvest quality

The identification of numerous marker-trait associations with fruit firmness and RDR suggests that postharvest quality in blackberries is a complex and highly polygenic trait. These findings are consistent with similar findings in a raspberry biparental linkage mapping study, which identified three large fruit firmness QTL across two different linkage groups (Simpson et al., 2017). Similarly, we report significant SNPs in three chromosomes, with Ra02 appearing to be the most important overall in determining postharvest quality. Both RDR and fruit firmness associations draw attention to a large network of texture-related homologs on Ra02 that are in high LD. This network is scattered across the chromosome and contains PG, PL, glucan endo-1,3-β-glucosidases, and expansins, which may all be synergistically contributing to postharvest quality. The association of both RDR and fruit firmness with this gene network may provide the genetic underpinning of the relationship previously reported between RDR and fruit firmness (Pérez-Pérez et al., 2018; Edgley et al., 2019b). The weakness of our observed phenotypic correlation between these traits is likely due to environmental influences impacting the phenotypic expression of RDR, but variation in anthocyanin composition between genotypes could also be a factor. The PME variant associated with fruit firmness on Ra01 was also located near a large cluster of 11 other texture-related homologs, providing further evidence of complex, polygenic control. Six of these genes were PME, suggesting that this locus could be important in the modification of pectin structure.

### Limitations of GWAS

One of the key strengths of a traditional GWAS performed across diverse panels of heterogeneous relatedness is in predicting common phenotypic variation with common genotypic variation (Myles et al., 2009). When important alleles are confined to a small number of individuals in the GWAS panel, detection of marker-trait associations becomes less likely. We expect this to be true in our own association analyses, considering the distribution of our phenotypic datasets. For instance, in our fruit firmness BLUP dataset, statistical outliers (1.5 x interquartile range) were present on both ends of the distribution, with A-2625T being the firmest and “Black Gem™” being the softest. Based on prior knowledge of these genotypes, these data seem valid. However, if these rare phenotypes are associated with rare underlying genotypes, statistical power will likely be insufficient to detect its source (Visscher et al., 2012). Such may be the case in detecting the cause of the rare ‘crispy’ texture phenotype documented in the UA System breeding program (Salgado and Clark, 2016), which has only been observed in a small number of genotypes to date. This texture is a distinct phenotype that is not easily recovered through crossing, and qualitative categorization of crispy genotypes is not easily achievable through the fruit firmness protocols employed in this study. For postharvest traits of unique importance like crispy texture, more targeted strategies such as biparental linkage mapping or RNA sequencing should be considered along with modified phenotyping protocols to explore its biological mechanism and modes of inheritance.

Another important consideration following a GWAS is in developing a genome-informed strategy for promoting long-term genetic gain in a breeding program. Our work suggests that postharvest quality is a complex trait that is likely governed by numerous genes on multiple chromosomes. In this context, the direct application of discovered QTL in MAS is unlikely to be sufficient. Instead, significant SNPs could be included in genomic selection models as fixed covariates based on their known importance (Rice and Lipka, 2019). In contrast with GWAS, genomic selection allows breeders to leverage the cumulative effects of many low-effect QTL in predicting the merit of new breeding selections. Furthermore, the large datasets accumulated in this study should provide an effective foundational training dataset for future genomic selection models. These data will allow breeders to predict blackberry fruit firmness and RDR susceptibility of genotypes and reduce the operational burdens of heavy phenotyping requirements. Such strategies are especially well-suited to complex and low-heritability traits like RDR and fruit firmness, which appear to be governed by numerous small-effect QTLs (Bernardo, 2016).

## Conclusion

This study is the first to report marker-trait associations in tetraploid blackberry, using a genome-wide approach. We have identified numerous SNPs significantly associated with RDR on Ra02 and seven SNPs associated with fruit firmness across four out of seven chromosomes. Variants on a PME located on Ra01, a glucan endo-1,3-β-glucosidase on Ra02, and a PG on Ra06 collectively explained 27% of the variance in fruit firmness in this study. A variant on a PG homolog on Ra02 accounted for 8% of the variance in RDR, but this chromosome also contained a much larger network of significantly associated variants on texture homologs in high LD. A cluster of six PME and PME inhibitor homologs were also identified within 500 kb of markers associated with fruit firmness on Ra01. Best linear unbiased predictions of RDR and fruit firmness were only weakly correlated in these populations, but overlapping genetic associations with both traits were discovered on Ra02, suggesting that potential candidate genes in this region may have pleiotropic effects. Given the modest heritability of fruit firmness and RDR, combined with their apparent polygenic nature, we determine that genomic selection is the most promising long-term strategy for improvement of these traits. Significant QTL should be used as fixed covariates in a genomic selection model that utilizes existing datasets for model training. These findings represent the first large-scale genomic exploration of a diverse blackberry population and will provide a strong methodological and conceptual framework for continued genetic research in the crop and its relatives.

## Data availability statement

The original contributions presented in the study are publicly available. This data can be found here: Genome Database for Rosaceae repository, accession number tfGDR1069.

## Author contributions

TC collected phenotypic data, performed bioinformatic and genetic analyses, and drafted the manuscript in collaboration with MW. MW wrote the grant to support this research, supervised graduate students and staff working on this project, and provided overall conceptual guidance. CJ and LN performed DNA extractions and quantification. JC developed most of the advanced selections and cultivars used in the study. RA and HA conducted bioinformatics to assist with Capture-Seq probe design. All authors contributed to the article and approved the submitted version.
