# Supplementary material for: Genome-wide association identifies key loci controlling blackberry postharvest quality
Source: Front Plant Sci. 2023 Jun 7;14:1182790. doi: 10.3389/fpls.2023.1182790 (PMC10282842; doi:10.3389/fpls.2023.1182790)
Supplement: Supplementary file 1 [file DataSheet_1.zip › Captions.docx]

Supplementary Materials

**Supplementary Tables**

Supplemental Table 1. Summary replicates evaluated, best linear unbiased predictors of red drupelet reversion and fruit firmness, and sequencing information for each of the 300 blackberry genotypes evaluated in the study.

Supplemental Table 2. Positions, LOD scores, gene names, and variant types of 220 SNPs significantly associated with red drupelet reversion.

**Supplementary Figures**

Supplemental Figure 1. The distribution of read depth across the blackberry 300 genotypes and 65,995 SNPs used in association analyses.

Supplemental Figure 2. The four steps of graphical determination of the true K(*) value as reported following Evanno et al. (2005). (A) Mean L(K) over three runs for each K value. (B) Rate of change in the likelihood distribution calculated as L’(K) = L(K) – L(K-1). (C) Absolute values of the second order rate of change in likelihood distribution calculated according to the formula: |L”(K)| = |L’(K + 1) – L’(K)|. (D) ΔK calculated as ΔK = m |L”(K)| / s[L(K)]. The modal value of this distribution is the true K(*).

Supplemental Figure 3. QQ plots of genetic marker p values across all 65,995 SNPs associated with fruit firmness tested under additive, general, and simplex dominance models.

Supplemental Figure 4. QQ plots of genetic marker p values across all 65,995 SNPs associated with red drupelet reversion tested under additive, general, and simplex dominance models.

Supplemental Figure 5. Heatmap of absolute values of Lewontin’s D’ (Lewontin, 1963) estimated between 220 SNPs significantly associated with red drupelet reversion on Ra02. Nonsynonymous variants of texture-related homologs are marked by blue triangles on the top edge of the heatmap matrix. The top line shows the relative physical position of markers at scale.
