# Supplementary figures and images for: Genome-wide association identifies key loci controlling blackberry postharvest quality
Source: Front Plant Sci. 2023 Jun 7;14:1182790. doi: 10.3389/fpls.2023.1182790 (PMC10282842; doi:10.3389/fpls.2023.1182790)

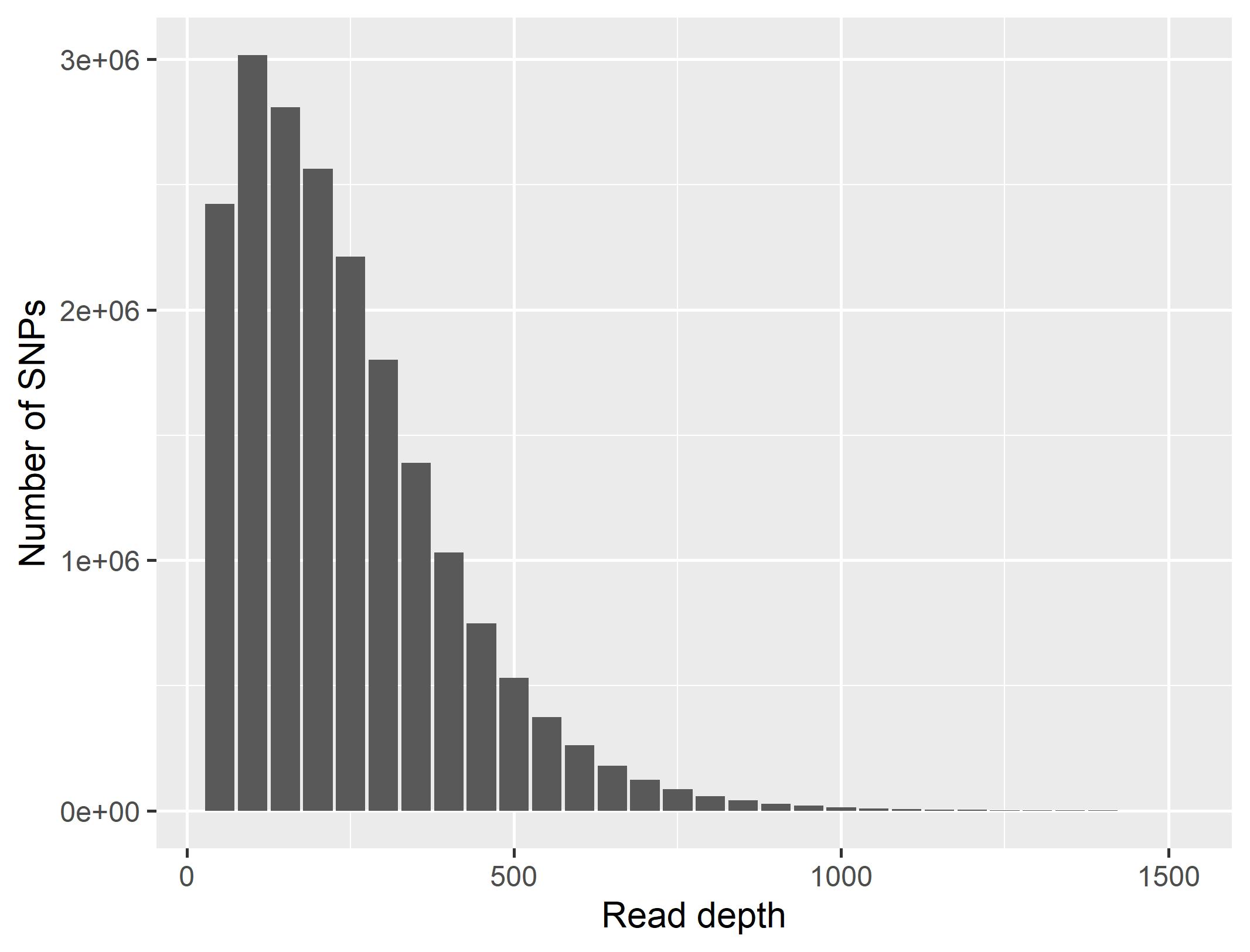

Supplement: Supplementary file 1 [file DataSheet_1.zip › Supplementary Figure 1.JPEG]

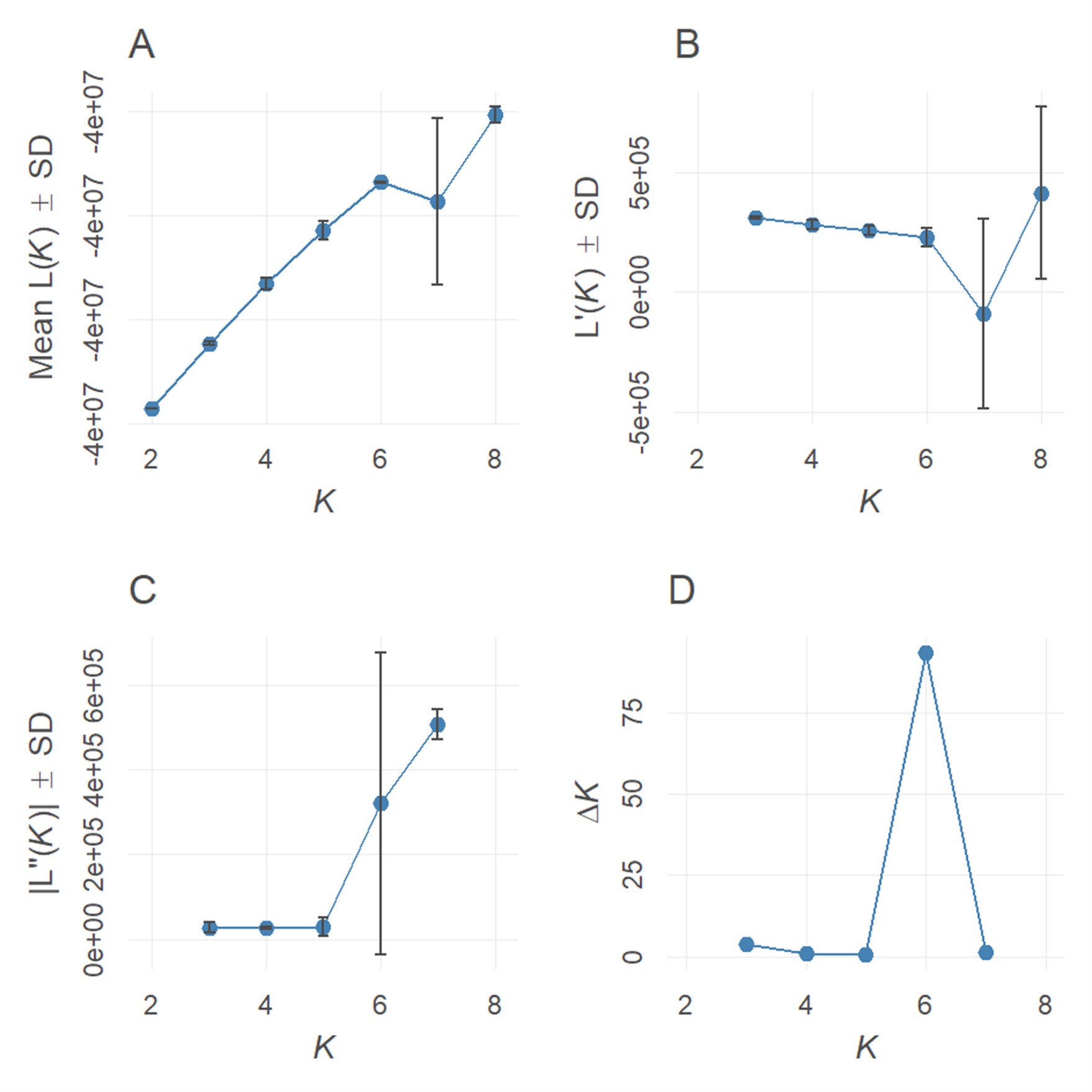

Supplement: Supplementary file 1 [file DataSheet_1.zip › Supplementary Figure 2.JPEG]

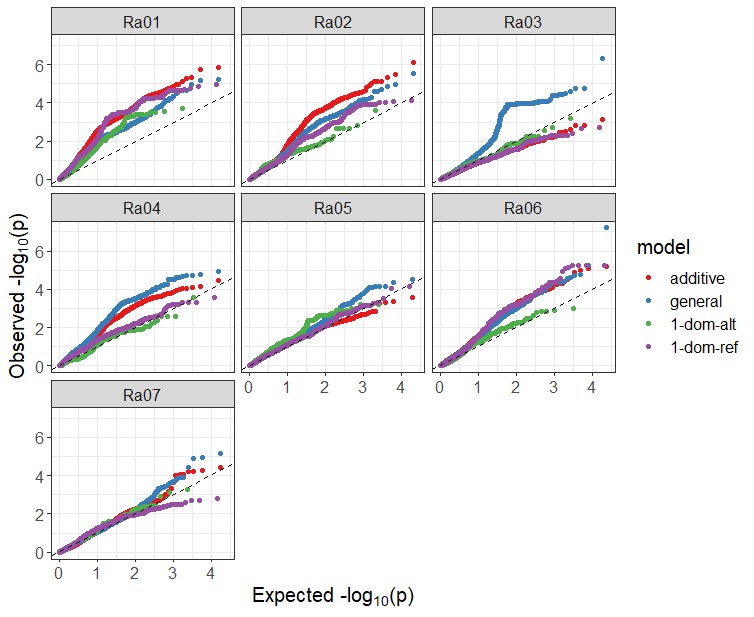

Supplement: Supplementary file 1 [file DataSheet_1.zip › Supplementary Figure 3.JPEG]

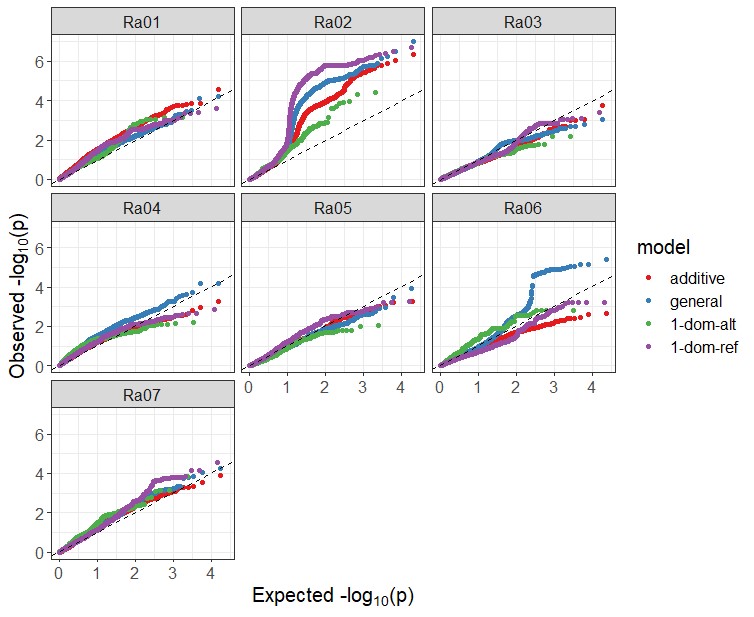

Supplement: Supplementary file 1 [file DataSheet_1.zip › Supplementary Figure 4.JPEG]

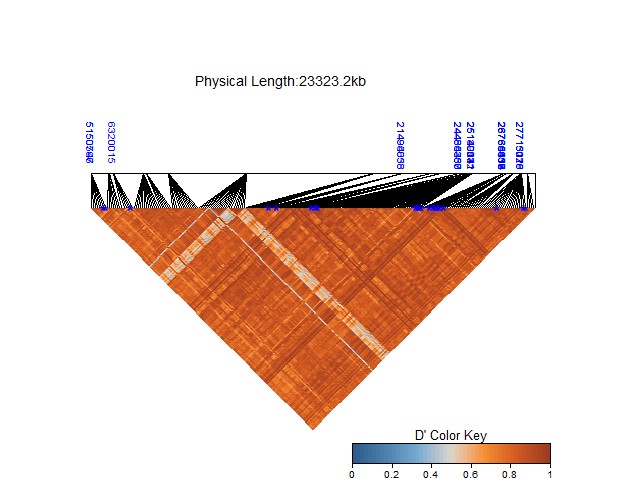

Supplement: Supplementary file 1 [file DataSheet_1.zip › Supplementary Figure 5.JPEG]
